# Supplementary material for: Proximity can induce diverse friendships: A large randomized classroom experiment
Source: PLoS One. 2021 Aug 11;16(8):e0255097. doi: 10.1371/journal.pone.0255097 (PMC8357142; doi:10.1371/journal.pone.0255097)
Supplement: S1 Text — (DOCX) [file pone.0255097.s001.docx]

Deviations Between the Pre-Analysis Plan and the Reported Analyses

We followed the pre-specified data collection and data exclusion procedures. We used the pre-specified primary outcome (reciprocated best-friend nominations) and primary treatment variables (assigned desk mate at baseline and the pre-specified desk-mate similarity index) to test the pre-specified Hypothesis 1 (desk mate exposure): The probability of a best-friend relationship increases if j is i’s deskmate. The following sections explain the rationale behind deviations between the pre-analysis plan and the reported analysis. There were two major deviations: we re-specified our models as multilevel models, and we omitted the test of a second hypothesis that raised some unexpected complexities. In the last section, we summarize and justify minor deviations.

Multilevel Models Instead of Social Relations Regression Models.

In the pre-analysis plan, we specified that we were going to test these hypotheses in a hierarchical social relations probit regression model. We had to adapt this plan for multiple reasons.

First, we had planned to use the R package *amen* by Peter D. Hoff. However, it turned out that *amen* is not suited to accommodate the hierarchical structure of the data. Hence, we re-specified our models in *brms* as described in the main text. These models are very similar on a conceptual level, except for omitting the particular covariance structure not relevant for the present research question.

To check whether our *brms* specification results in similar results as the originally planned *amen* implementation, we ran both analyses for a random subset of 50 classrooms, applying the models to one classroom at a time. The point estimates of the deskmate effect were highly correlated, *r* = .831. While for most classrooms, models returned plausible estimates, some classrooms resulted in very large negative point estimates with considerable uncertainty (i.e., for 24% of the classrooms, effects were estimated to be smaller than -2). This has to do with the friendships within the respective classrooms: If *none* of the deskmate dyads reports a reciprocated friendship, the estimate would become negative infinity were it not for the regularizing influence of the prior. Because of differences in the model priors, these outliers (here defined as coefficients below -2) were much more extreme in *brms* (mean among outliers -234.01) than in *amen* (mean among outliers -4.08). When ignoring these outliers, the correlation between the estimates was almost perfect (*r* = .992) and the absolute differences in the deskmate probit coefficients between *brms* and *amen* were small (*M_absolute difference_* = 0.06), indicating that both types of models lead to highly similar conclusions except for cases in which the data situation made it impossible to give a reliable estimate of the deskmate effect.

Notice that the negative outliers no longer appear in the combined overall analysis in *brms*, even when deskmate effects were allowed to vary by classroom (Figure S1): The nature of the multi-level model provides regularization, shrinking estimates in classrooms that do not allow for a reliable estimation of the effect towards the average effect.

Omission of Hypothesis 2

The pre-analysis plan specified another hypothesis, which has been omitted from this manuscript, Hypothesis 2 (similarity between class mate and desk mate): The probability of a best-friend relationship increases if classmate j resembles student i’s desk mate. There are multiple complications with the model we had planned to test this hypothesis which eventually led us to exclude it from this manuscript.

First, somewhat trivially, the model in the pre-analysis plan was redundantly notated. The pre-specified analysis would have included (1) whether or not i and j were seated next to each other, (2) the similarity between i’s deskmate and student j (labelled “deskmate similarity”), and (3) the interaction of the two. But if i and j are deskmates, then the similarity between i’s deskmate (j) and student j will be 1 and the interaction term is 1. If the two students are not deskmates, the interaction term is 0. In other words, the interaction term is fully redundant with predictor (1). This issue is an inconsequential but unnecessary notational redundancy.

Furthermore, predictor (2), deskmate similarity, is actually causally affected by the deskmate variable (1). Hence, including (2) will distort the estimate of the effect of (1), the central treatment of interest. Therefore, even though we had planned to test both hypotheses within a single model, when testing Hypothesis 1, deskmate similarity ought not to be included or else the deskmate effect is no longer causally identified.

Apart from these issues, we discovered that even after correcting the model for the above noted endogeneity, the pre-registered model would falsely detect arbitrary “effects” in a simulated Null model. Fixing this issue will require methodological work that would go beyond the present manuscript. Since we regard the deskmate hypothesis as the main substantive point of interest in this study, we elected to reserve the other hypothesis for future work.

Smaller Deviations

**Liking of Deskmate.** The pre-analysis plan stated in the section Secondary Outcomes: “Second, we will analyze how much student I liked their desk mate, coded 1-5, where 1 is best and 2 is least and ‘don’t know’ is coded as ‘neutral’ = 3 (Question 7).” While this variable has been collected, its utility to address any substantive questions is questionable, because this question has only been asked with respects to deskmates, not with respects to other students in the classroom—hence, it cannot be used as an alternative outcome of the deskmate intervention.

**Missing values.** The pre-analysis plan states that “If we have missing values on a covariate we will code the covariate as zero and include dummy variables controlling for missing status so that we do not lose observations.” This approach is controversial from a methodological perspective. Furthermore, the relevant variables for which we observe missing values (ethnicity, GPA) are never just used for the purpose of covariate adjustment but are instead central predictors of interest. Therefore, and because there were only few missing values (5.5% of dyads lacked information on ethnicity, 6.2% of dyads lacked information on GPA), we instead simply excluded dyads for which the relevant variables were missing.
