# Supplementary material for: Proximity can induce diverse friendships: A large randomized classroom experiment
Source: PLoS One. 2021 Aug 11;16(8):e0255097. doi: 10.1371/journal.pone.0255097 (PMC8357142; doi:10.1371/journal.pone.0255097)
Supplement: S2 Text — (DOCX) [file pone.0255097.s002.docx]

Details Regarding Pre-Treatment Variables

From a survey of classroom teachers (*N* = 160) prior to the intervention, we know that (a) 74% of teachers design the seating chart in their classrooms, (b) some teachers prefer to assign high and low ability students (48.8%) and well and badly behaved students (41.3%) to the same desk. According to teachers’ answers students’ gender and ethnicity are not important dimensions of the seating chart: 75% and 95.6% of teachers reported that these characteristics do not play a role in designing the seating chart. We do not have information on prior deskmate relationship of these students; this information would have allowed us to ask additional questions, but it is not necessary to justify the claims we make in this study—the causal effects we estimate are identified by randomization.
