# Supplementary material for: Proximity can induce diverse friendships: A large randomized classroom experiment
Source: PLoS One. 2021 Aug 11;16(8):e0255097. doi: 10.1371/journal.pone.0255097 (PMC8357142; doi:10.1371/journal.pone.0255097)
Supplement: S3 Text — (DOCX) [file pone.0255097.s003.docx]

Balance Checks

Following our preregistration plan, we tested for balance following Guryan, Kroft, and Notowidigdo (2009) by regressing each student’s own baseline characteristic (gender, ethnicity, GPA based on the five reported grades) on (1) the student’s deskmate’s baseline characteristic, (2) the leave-one-out mean characteristic in the classroom, and (3) classroom fixed effects. This procedure corrects for the artifactual correlation between students’ and their deskmates’ characteristics induced by randomly partitioning students to desks within classrooms (see e.g. Boozer & Cacciola, 2001). Standard errors were clustered at the school-level. Results indicated excellent balance, with no significant associations between students’ and deskmates’ GPAs (*p* = .311), gender (*p* = .558) or ethnicity (*p* = .886).
