# Supplementary material for: Proximity can induce diverse friendships: A large randomized classroom experiment
Source: PLoS One. 2021 Aug 11;16(8):e0255097. doi: 10.1371/journal.pone.0255097 (PMC8357142; doi:10.1371/journal.pone.0255097)
Supplement: S1 Table — (DOCX) [file pone.0255097.s004.docx]

**S1 Table**

Results of Bayesian multi-membership multilevel probit models investigating the modifying role of single dimensions of similarity.

|  | Gender | | Educational Achievment | | Ethnicity | |
| --- | --- | --- | --- | --- | --- | --- |
|  | Estimate | 95% CI | Estimate | 95% CI | Estimate | 95% CI |
| σ_Student_ | 0.48 | [0.41; 0.54] | 0.04 | [0.00; 0.10] | 0.04 | [0.00, 0.11] |
| Reference group | Both boys | |  |  | Both Non-Roma | |
| β_0_ | -0.47 | [-0.82; -0.15] |  |  | -0.94 | [-1.15; -0.75] |
| β_Deskmate_ | 0.39 | [0.07; 0.24] |  |  | 0.30 | [0.21; 0.40] |
| β_mixed Dyad_ | -1.70 | [-1.78; -1.63] |  |  | -0.21 | [-0.28; -0.14] |
| β_concordant Dyad_ | 0.10 | [0.03; 0.16] |  |  | -0.08 | [-0.18; 0.03] |
| β_mixed Dyad*Deskmate_ | 0.01 | [-0.23; 0.26] |  |  | -0.18 | [-0.42; 0.04] |
| β_concordant Dyad*Deskmate_ | -0.12 | [-0.32; 0.09] |  |  | -0.17 | [-0.41; 0.07] |
| β_Mean GPA_ |  |  | 0.14 | [0.11; 0.16] |  |  |
| β_Mean GPA*Deskmate_ |  |  | -0.02 | [-0.10; 0.07] |  |  |
| β_GPA difference_ |  |  | -0.19 | [-0.22; -0.17] |  |  |
| β_GPA difference*Deskmate_ |  |  | -0.01 | [-0.10; 0.07] |  |  |
| N_Dyads_ | 24,962 | | 23,408 | | 23,594 | |
| N_Students_ | 2,996 | | 2,996 | | 2,860 | |

Concordant dyad refers to dyads consisting of two boys or dyads consisting of two Roma students respectively. Cells relevant for the assessment of the modification-by-similarity hypothesis on the probit scale are shaded.
