# Supplementary figures and images for: Proximity can induce diverse friendships: A large randomized classroom experiment
Source: PLoS One. 2021 Aug 11;16(8):e0255097. doi: 10.1371/journal.pone.0255097 (PMC8357142; doi:10.1371/journal.pone.0255097)

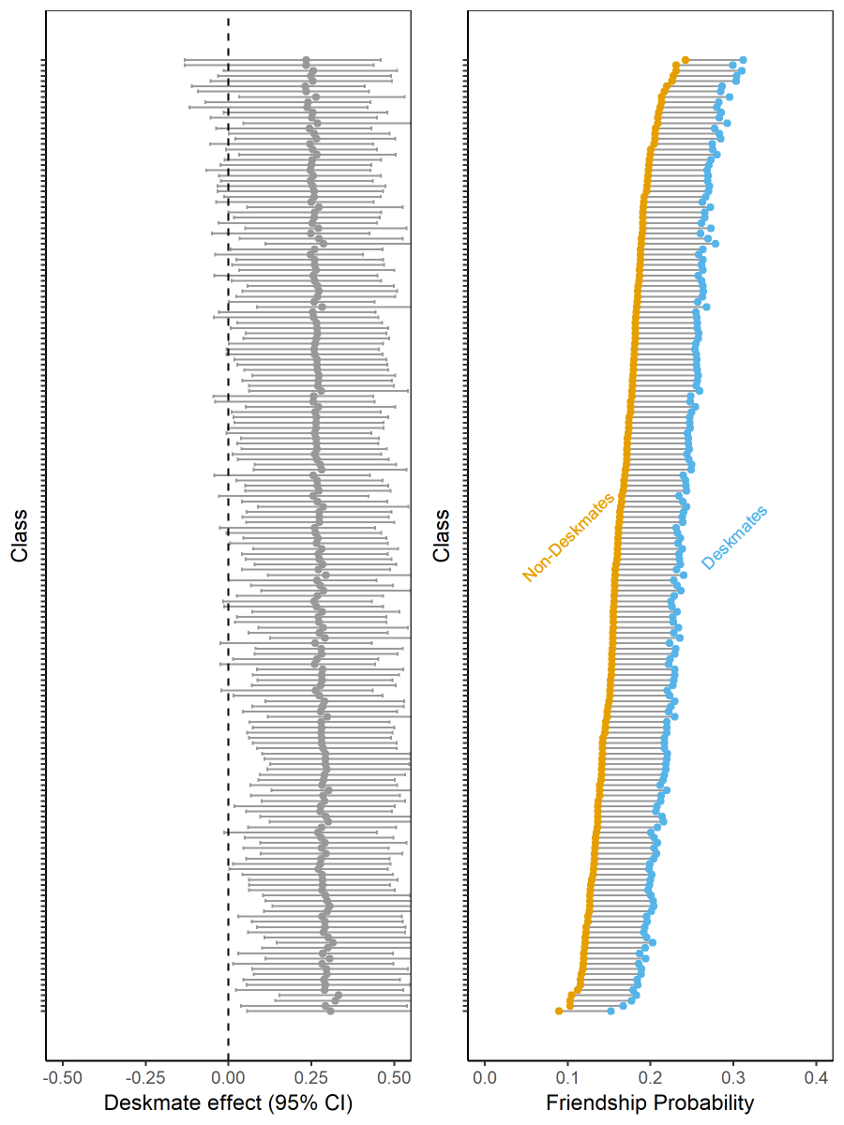

Supplement: S1 Fig — Probit coefficients from random effects model (left panel) as well as the corresponding model-implied friendship probabilities for non-deskmates versus deskmates (right panel). The difference between each predicted probability for deskmates minus the predicted probability for non-deskmates is the classroom-specific AME. (TIF) [file pone.0255097.s005.tif]
