# Supplementary material for: Proximity can induce diverse friendships: A large randomized classroom experiment
Source: PLoS One. 2021 Aug 11;16(8):e0255097. doi: 10.1371/journal.pone.0255097 (PMC8357142; doi:10.1371/journal.pone.0255097)
Supplement: Attachment — Submitted filename: Response to Reviewers V6.docx [file pone.0255097.s006.docx]

**Point-by-point response**

**Editorial remarks:**

**E.1. I agree with Reviewer #2 that you should provide, if available, ex-ante information on pre-treatment students’ friendship and pre-treatment seating rules. This piece of information is crucial especially for pupils in higher grades.**

We have revised the manuscript to clarify that teachers typically design the seating chart. We provide additional information on typical seating practices in our response to R.2.2 below.

We do not have ex-ante information students’ prior friendships or past seating charts. We agree that information on prior friendships (i.e., lagged outcomes), or prior deskmates (i.e., lagged treatments) would have been interesting, as it would have allowed us to answer additional research questions, and may potentially have increased efficiency.

However, the absence of this information is not a problem for the causal claims we make: We are able to identify the causal effects of having a particular deskmate because we randomized the seating chart. These causal effects are the central focus of our manuscript. Thus, believe that our study makes valuable contributions since it is the first large randomized experiment on the effect of spatial proximity within classrooms on friendships.

**E.2. Furthermore, you specify in the paper that the students were randomly seated in three main subjects, but it is not clear how you chose these subjects and, most importantly, what happens during the other subjects.**

Thank you for this suggestion! Indeed, this is important background information. We have expanded the relevant manuscript section to include it.

p. 8, starting from line 163: Teachers were instructed to employ the intended seating chart in the three core subjects of the curriculum—mathematics, Hungarian literature, and Hungarian grammar—from the first day of classes (September 1, 2017) until the end of the fall semester (January 31, 2018). These three subjects form the core of the curriculum and receive the greatest weight in admission to selective secondary schools. They were taught in the same room for all grade levels and accounted for 6 to 10 lessons per week (25 to 45 percent of all lessons). Enforcing the seating chart across all subjects was not possible because (1) in some subjects, classrooms were split into smaller groups (e.g., different foreign languages), and (2) in some subjects, students were not seated in a fixed grid-layout (e.g., physical education and arts). However, seating charts typically apply to all subjects in a given room, and (depending on the grade level) most subjects were taught in the same room. Thus, students assigned to sit next to each other in the three core subjects likely also sat next to each other in other subjects; but we did not verify adherence outside of the core subjects.

The decision to limit the intervention instructions to the three core subjects was thus made for the sake of practicality. (We expect that non-adherence to the seating-chart in other subjects would, if it were random, dilute the effects of our intervention and render our estimates conservative. However, since we have no formal result for this expectation, we chose not to include it in the manuscript.)

**E.3. I also recommend to discuss more in detail the issues of unobservable teacher characteristics and sample selection raised by the same reviewer.**

We address this question below in response to R.2.4, in order to present our answer in the context of the verbatim reviewer comment.

**E.4. Finally, as suggested by Reviewer #1, it may be interesting to investigate the existence of heterogeneous effects by class size. Regarding heterogeneous effects already discussed in this version of the paper, the reviewer provides a number of suggestions to clarify your results.**

Done! Please see our detailed response below (R.1.3).

**E.5. If applicable, we recommend that you deposit your laboratory protocols in protocols.io to enhance the reproducibility of your results.**

We are commited to research transparency and reproducibility. We were unaware of the protocols.io platform, which seems like a great fit for laboratory research in particular. For the present project, we have already made all relevant information available on the Open Science Framework (<https://osf.io/4vjc5/?view_only=d0e9a887b3da4ebcabd0f9afb7480d65>). We hope this is acceptable.

**E.6. Please ensure that your manuscript meets PLOS ONE’s style requirements, including those for file naming.**

We have carefully checked all materials to ensure that they adhere to the style requirements.

**E.7. Please provide additional details regarding participant consent. In the ethics statement in the Methods and online submission information, please ensure that you have specified what type you obtained (for instance, written or verbal, and if verbal, how it was documented and witnessed). If your study included minors, state whether you obtained consent from parents or guardians. If the need for consent was waived by the ethics committee, please include this information.**

Done! We have provided the following details regarding participant consent in the revised manuscript:

p. 16, line 353: This study was reviewed and approved by the IRB offices at the Centre for Social (data collection and analysis), and at the University of Wisconsin-Madison (data analysis). Consent was obtained at multiple points. School districts, school principals, and teachers provided written consent to participating in the seating chart randomization. Parents provided written consent for the retrieval of administrative records via teachers, and for their children’s participation in the survey.

**E.8. We note that you have stated that you will provide repository information for your data at acceptance. Should your manuscript be accepted for publication, we will hold it until you provide the relevant accession numbers or DOIs necessary to access your data. If you wish to make changes to your Data Availability statement, please describe these changes in your cover letter and we will update your Data Availability statement to reflect the information you provide.**

We are fully committed to provide the data and all other relevant information on the Open Science framework. All materials can already be accessed under <https://osf.io/4vjc5/?view_only=d0e9a887b3da4ebcabd0f9afb7480d65>. We will make the corresponding project public upon acceptance.

**Reviewer #1**

**This paper reports results from a field experiment conducted on a sample of Hungarian pupils enrolled in primary school. It shows that exogenous induced physical proximity might increase the likelihood of friendship formation. This effect seems stronger when the physical proximity is induced on students with a higher degree of “similarity”.**

**I find this paper quite interesting and generally well-executed. Overall, I see the potential for publication. I have just a couple of comments, which are reported below.**

Thank you for this positive feedback.

**R.1.1. I would suggest moving the section “Modification-by-Similarity Hypothesis: Moderating Influence of Gender, Educational Achievement and Ethnicity” before section “Modification-by-Similarity Hypothesis: Moderating Role of Overall Similarity”. I feel it is better to understand first the characteristics driving the effects’ heterogeneity and then an overall assessment via a comprehensive measure of similarity.**

We see the merit of this suggestion—the “bottom-up” logic (start with single dimensions, then aggregate) may be easier to follow for some readers. On the other hand, our pre-analysis plan states that the combined index of similarity is of primary interest; the single dimensions are only listed as secondary (exploratory) analyses. Thus, we have respectfully elected to keep the current structure in order to to honor the pre-registered sequence of primary vs. secondary analyses. We thank the reviewer for this thoughtful idea.

**R.1.2. Looking at the results for each dimension of similarity separately, it seems that gender is the only characteristic that matters in altering the primary effect of being a deskmate. Indeed, the grouped GPA and ethnicity marginal effects are never statistically significantly different from each other (Fig 1, H and F). This aspect should also be clarified in the text. For instance, the last sentence in the abstract gives the impression that ethnicity seems to be a relevant character but is not that clear from the results.**

We agree with the reviewer’s interpretation that gender is the main driver of modification by similarity (on the average-marginal-effect [AME] scale). As for the other two dimensions, the story is somewhat complicated. For example, the pairwise comparisons of the effect by GPA-category may not be statistically significant in themselves, but they align with the trend higher similarity 🡪 larger AME; hence this GPA trend will still contribute to the modification by overall similarity.

As requested, we have sharpened the prose, including in the abstract, to be crystal clear.

Abstract: […] the probability of a manifest friendship increased more among similar than among dissimilar students—a pattern mainly driven by gender. Our findings demonstrate that a scalable light-touch intervention can affect face-to-face networks and foster diverse friendships in groups that already know each other, but they also highlight that transgressing boundaries, especially those defined by gender, remains an uphill battle.

p. 22, line 491: But since the effect of a given increase in the latent propensity toward friendship on the formation of a manifest friendship also depends on the dyad’s baseline propensity toward friendship, and since more similar dyads have a greater baseline propensity toward friendship (homophily), the intervention was more successful at inducing manifest friendships among similar students than among dissimilar students. The three dimensions of similarity that we investigated contributed to this pattern to varying degrees: Gender showed the clearest effect modification (smaller effects among mixed-gender dyads), with a weak but aligned trend for baseline GPA (smaller effects when grade differences were large), and a somewhat misaligned trend for ethnicity (smaller effects in mixed *and* in Roma dyads).

**R.1.3. With the class fixed effects, you control for the unobservables related to the class. However, could you highlight how the effect changes depending on the size of the class-size? Is the effect stronger in larger classes? It should be appropriate to run an interaction model with an indicator for sample size (for instance, being in the top or bottom tertile of class-size distribution), but given how the results are presented, it could also work a sample splitting.**

Thank you for suggesting this interesting additional analysis. We derived the requested estimates from the model in which the effect was allowed to vary freely from classroom to classroom. These exploratory findings are now briefly summarized in the manuscript:

p. 17, line 381: In models in which we allowed the deskmate effect to vary between classrooms, we found some variability of the deskmate effects, but the differences were substantively small (*SD_Deskmate effect_* = 0.09, $CI_{95}$: [0.00, 0.23]), see S3 Fig. These models also suggested that the number of students in the classroom did not modify the effect (bottom tertile, 17 students or fewer: *AME* = 7.5 percentage points, $CI_{95}$: [4.5, 10.5], top tertile, more than 20 students: *AME* = 7.3 percentage points, $CI_{95}$: [4.4, 10.5]).

**Reviewer #2**

**R.2.1. I have some concerns on the design:**

**- ex-ante information: we miss information on students’ ex-ante friendship relationship within the class. This would have allowed a much cleaner design and test of the research hypotheses. Do the authors have such information?**

This is a great question. Unfortunately, we do not have information on prior friendships within classes. While such information would have allowed us to ask *additional* questions and possibly have increased power (maybe this is what the reviewer means by a “cleaner test”), we respectfully submit that this information is not necessary to justify the claims we make in this study. Randomization of the seating chart on its own is sufficient to cleanly identify the causal effect of deskmates on friendship formation. (See also response to E1).

**R.2.2. how are students’ seat decided usually? Do students choose? Are they in alphabetical order? Do the authors have information on the previous seating scheme within each class?**

From a survey of classroom teachers (*N* = 160) prior to the intervention, we know that (a) 74% of teachers design the seating chart in their classrooms, (b) some teachers prefer to assign high and low ability students (48.8%), or well and badly behaved students (41.3%), to the same desk. According to teachers’ answers, students’ gender and ethnicity are not important considerations when desiging the seating chart: 75% and 95.6% of teachers reported that these characteristics do not play a role in designing the seating chart. We do not have information on prior deskmate relationship of these students; this information would have allowed us to ask additional questions, but it is not necessary to justify the claims we make in this study—the causal effects we estimate are identified by randomization.

We have revised the body of the text to include that (p. 4, line 91) “ordinarily, the majority of seating charts would be designed by teachers, see S2 Text.” S2 Text contains the entire paragraph above.

**R.2.3. why do the authors choose only three subjects? I imagine they are the subjects who represent most of the teaching hours. But, wouldn’t be better to ask to fix students’ seat for all the teaching subjects? It is not clear what happens during the other subjects. Do students change their seating when the subject changes?**

This is an important question—and one that we pondered extensively during the design phase of the study.

We fully agree that it would have been optimal to fix students’ seating charts for all subjects. But this was not feasible in practice, given that students sometimes change rooms for different subjects.

We now provide this additional information in the revised manuscript:

p. 8, starting from line 163: Teachers were instructed to employ the intended seating chart in the three core subjects of the curriculum—mathematics, Hungarian literature, and Hungarian grammar—from the first day of classes (September 1, 2017) until the end of the fall semester (January 31, 2018). These three subjects form the core of the curriculum and receive the greatest weight in admission to selective secondary schools. They were taught in the same room for all grade levels and accounted for 6 to 10 lessons per week (25 to 45 percent of all lessons).. Enforcing the seating chart across all subjects was not possible because (1) in some subjects, classrooms were split into smaller groups (e.g., different foreign languages), and (2) in some subjects, students were not seated in a fixed grid-layout (e.g., physical education and arts). However, seating charts typically apply to all subjects in a given room, and (depending on the grade level) most subjects were taught in the same room. Thus, students assigned to sit next to each other in the three core subjects likely also sat next to each other in other subjects; but we did not verify adherence outside of the core subjects.

**R.2.4. selection: it can happen at different levels. First, line 127, recruitment depends on teachers’ decision to implement the protocol. Teachers deciding to take part to the study may have unobserved characteristics that also influence pupils attitudes towards - let’s call them - “several kinds“of friendship. Second, some schools are then dropped because they did not meet the inclusion criteria; third students and schools are dropped from the sample because they did not answer the friendship-nomination item which is used to create the outcome variable. The authors should show that selection is not an issue. The first step in this direction would be to compare observable characteristics across samples.**

We believe that this comment addresses multiple types of selection (cf. Imai, King, Stuart. 2008—“Misunderstandings between experimentalists and observationalists about causal inference” JRSS-A).

Points 1 (teachers) and 2 (schools) concern “selection” on baseline characteristics. Selection on baseline characteristics is regrettable but par for the course in field experiments, which, to an extent, rely on subjects’ willingness to participate in an intervention. This type of selection only concerns external validity (i.e., generalizability or transportability of results across contexts). It does not threaten the internal validity, i.e., identification of causal effects within the study sample. The trade-off between internal and external validity in favor of achieving internal validity is standard in field experiments (Imai et al. 2008), although it goes without saying that it would be preferable to have both (Imai et al. 2008).

Selection on the outcome (friendship nominations), had it occurred, by contrast, would additionally threaten internal validity (i.e., identification). We are optimistic that this problem, should it exist, is minor in our study. First, we emphasize that all exclusion criteria were pre-registered prior to the receipt of outcome data. This prevents us from “fishing” for desired results. Second, outcome data are missing only for a small share of the sample (391 students, 10.3% of the pre-registered 3,814 students), a share that is in line with other well-regarded randomized field experiments. Third, most missingness in the outcome is owed to lack of parental consent to participate in the endline survey, rather than due to item non-response. One would have to craft very elaborate scenarios to link parental lack of consent to bias in the main analysis. Specifically, it would have to be the case that parental consent for the endline survey is a function of the outcome, i.e., whether or not their child had befriended their deskmate (and even in such a scenario, our analysis would be a valid test of the null hypothesis of no effect). That is not to say that we can rule out any threat of bias; only that we do not think this problem is of special concern for our study.

**R.2.5. can the authors check the robustness without students with self reported measures?**

We assume that this question referrs to the fact that, following the pre-analysis plan, missing teacher reports of baseline covariates were filled in with students’ self-reports collected at endline (line 189). Note that this decision was pre-registered, and it only affected a small number of students (depending on the subject, between 3.2 and 3.6% of the grades were filled in from self-reports). Nonetheless, to ensure that our results weren’t sensitive to this decision, we re-ran analyses limiting the sample to students for which teacher reports were available.

In brief, results were highly similar. The estimated average marginal effect of the intervention was 7.2 percentage points, 95% CI: [4.8; 9.6] as opposed to 7.0 percentage points, 95% CI: [4.6; 9.4]. Considering effect modification by similarity, once again results were virtually unchanged, for example: AME among low similarity students 1.7 [0.3; 3.3] as compared to 1.5 [0.2; 3.1]; among average similarity students 5.9 [3.5; 8.3] as compared to 5.7 [3.4; 8.0]; among high similarity students 11.6 [7.8; 15.6] as compared to 11.8 [8.0; 15.7].

We can thus be confident that the decision to rely on student self-reports (where necessary) did not affect conclusions. The full analytic output can be found on the Open Science Framework: https://osf.io/sbn6h/.

**R.2.6. line 149: have you checked the robustness of results without such 5.6%**

We apologize for not understanding this question. Neither on Line 149, nor anywhere in the manuscript or supplement do we refer to the number “5.6.” In the supplemental text, we state that 5.5% of dyads lacked information on ethnicity. These dyads have already been excluded from analysis (as stated in the supplement).

**R.2.7. line 329: can the authors comment on the size of the effect? 1.6 compared with 7 percentage points seems quite a relevant difference**

We believe this to be a misunderstanding—1.6 compared to 7 percentage points would indeed be quite relevant. However, as we state in the manuscript, this “1.6” refers to the largest *discrepancy* between estimates. We have clarified the phrasing, line 346: “The largest absolute difference in the estimated AMEs across models was 1.6 percentage points”

To provide the full context for this largest observed difference:

In the focal models (specification as reported in the manuscript), we estimate that the deskmate effect among boy-dyads is 41.28 – 28.14 = 13.14 percentage points (numbers rounded, more precise output reported on the OSF). For gender-mixed dyads, the deskmate effect is 4.02 - 1.69 = 2.33 percentage points. The estimated difference between these deskmate effects is 10.8 percentage points, 95% Credible Interval: 5.48, 16.15.

In our linear probability models, these estimates look slightly different. The deskmate effect among boy-dyads is 42.06 – 27.88 = 14.18 percentage points; among gender-mixed dyads it is 2.94 – 1.15 = 1.80. The estimated difference between these deskmate effects is 12.4 percentage points, 95% Credible Interval: 7.85, 17.10.

The difference between the differences estimated by the two model specifications—12.4 percentage points versus 10.8 percentage points—is 1.6 percentage points, the largest observed discrepancy. We think that this deviation is rather unsurprising given that (1) we would expect the probit model and the linear probability model to behave differently close to zero and given that (2) differences in differences are estimated with rather large uncertainty (see wide credible intervals).

Hence, this small discrepancy does not affect our qualitative conclusions.

**R.2.8. the authors can include equations of the estimated models and tables with the estimation results. This would help to understand their econometric technique and make the reading of the paper more fluent**

Thank you for this suggestion. We have revised the manuscript to state the main Bayesian multi-membership multilevel model in standard econometric notation, see page 10:

We model the effect of sitting next to each other on reciprocated friendship nominations using a Bayesian multi-membership multilevel probit model. This is a dyad-level model with one observation for each unordered dyad consisting of students $i$ and $j$ in classroom $c$,

${Friendship}_{\{ij\}c}^{*}=\beta_{0}+\beta_{D}*{Deskmate}_{\{ij\}c}+{Classroom}_{c}+\sum_{s\in\{i, j\}} {Student}_{sc}+\epsilon_{\{ij\}c}$ (1)

where $Friendship_{\{ij\}c}^{*}$ is the latent continuous friendship propensity of the dyad; ${Deskmate}_{\{ij\}c}=1$ if the students in the dyad are deskmates and $=0$ otherwise; $Classroom_{c}$ is a vector of classroom fixed effects to account for randomization within classrooms; and $\epsilon_{\{ij\}c}\sim N(0, \sigma_{\epsilon}^{2})$ is the i.i.d. dyad-specific error term. The term $\sum_{s\in\{i, j\}} {Student}_{sc}$ refers to the two i.i.d. random effects for the students $s$ in the dyad, $Student_{sc}\sim N\left( 0,\sigma_{Student}^{2} \right)$. The latent continuous friendship propensity is linked to manifest friendship via the threshold function $Friendship_{ijc}=1$ if $Friendship_{ijc}^{*}>0$ and $=0$ otherwise.

Furthermore, we added a table with estimation results of the two primary analyses. We also added S5 Table with estimation results of the follow-up analyses of the single dimensions of similarity.

Table 1. Results of Bayesian multi-membership multilevel probit models for the effects of sitting next to each other on reciprocated friendship.

|  | Main Analysis | | Modification by Overall Dyadic Similarity | |
| --- | --- | --- | --- | --- |
|  | Estimate | 95% CI | Estimate | 95% CI |
| b_0_ | -0.96 | [-1.20; -0.72] | -1.49 | [-1.84; -1.14] |
| b_Deskmate_ | 0.27 | [0.19; 0.35] | 0.29 | [0.19; 0.39] |
| σ_Student_ | 0.04 | [0.00; 0.10] | 0.52 | [0.46; 0.58] |
| b_Similarity_ |  |  | 0.83 | [0.79; 0.86] |
| b_Similarity*Deskmate_ |  |  | 0.07 | [-0.05; 0.18] |
| N_Dyads_ | 24,962 | | 24,962 | |
| N_Students_ | 2,996 | | 2,996 | |

This table lists probit coefficient. Average marginal effects were derived from the fitted probit model following the procedure described in the Methods section. We decided to retain the figure presenting the AMEs and believe that this dual-presentation strategy (Table and Figure) offers a good balance for readers from a broad variety of fields.

**R.2.9. Please, spellcheck the paper because I have spotted some typos: i.e. line 154 “students“; line 155 “with“; line 250 “be friend“; line 461 “replicating”**

Thank you for your careful reading of our manuscript! We have fixed the mistakes in (original) line 154, 155, and 461. Our use of the intransitive verb “to befriend” in line 250 is correct. We have given the entire manuscript another careful read to correct remaining mistakes.
