# Supplementary material for: Proximity can induce diverse friendships: A large randomized classroom experiment
Source: PLoS One. 2021 Aug 11;16(8):e0255097. doi: 10.1371/journal.pone.0255097 (PMC8357142; doi:10.1371/journal.pone.0255097)
Supplement: Attachment — Submitted filename: Response to Reviewers_V3.docx [file pone.0255097.s007.docx]

**Editorial Remarks**

**E.1. The main issue that can threat your identification strategy remains self-selection, which may be influenced by treatment status. The reviewer provides an illuminating example on this. If you do not have data to provide further robustness checks on this issue, you should at least discuss whether and how this may influence your estimates.**

Reviewer 2 brings up two potential issues of selection: (1) study participation (i.e., selection prior to baseline which does not threaten internal validity but may affect external validity) and (2) selective attrition (i.e., selection after baseline), which may threaten internal validity.

Concerning (1), we added a paragraph to the body of the text that explicitly acknowledges potential threats to external validity, see response to R.2.2.

Concerning (2), we ran extensive additional analyses to investigate and address the matter. Despite low levels of attrition, we did indeed find some evidence for selective attrition, as suggested by the reviewer. We thus ran two additional sets of analyses: lower bound analyses in which missing responses were imputed under the most conservative assumptions; and analyses incorporating non-response weights. Both resulted in somewhat lower point estimates than the 7.0 percentage-points estimate in our pre-registered primary analyses of complete cases: our lower bound estimate is 4.0 percentage points, and our non-response weighted estimate is 6.1 percentage points. In both cases, the 95% CI still comfortably excludes zero. More details can be found in our response to R.2.3. We have adjusted the language throughout the manuscript at multiple points to acknowledge these new and smaller estimates (see also response to R.2.3).

**E.2. Similarly, you should discuss more clearly the role of pre-existing friendships in driving your results.**

Thanks to the helpful clarification by the reviewer, we now understand that the reviewer’s remark does not relate to the identification of the causal deskmate effect, which is the focus of our study, but to the interpretation of the role of similarity, which is an effect modifier (i.e., it indexes effect heterogeneity in the causal deskmate effect). In short, the interpretation implied by the reviewer is fully compatible with our interpretation (and we added it to the manuscript), see response to R.2.4. for details.

**Reviewer #2**

**R.2.1. I think that the authors have done a good job in addressing the reviewers’ comments and the revised version of the paper is much clearer.**

Thank you very much!

**R.2.2. About selection: first, I think that, if the authors do not want or can show the observable characteristics of the teachers and schools that selected out of the study, at least they should discuss the external validity of the study**

We did not collect extensive data on the teachers and schools who were asked to participate in the study and thus cannot provide a comprehensive analysis of potential limitations of external validity. However, we have school-level data of the schools who decided to participate, which we can compare to all other Hungarian primary schools to give a better idea of the setting of the study.

Of course, the limited external validity of experimental studies always merits a reminder, and so we added a paragraph to the discussion:

p. 24, line 552: “It remains, of course, an open question whether our findings generalize to other settings and countries. Our study took place in a less prosperous area of rural Hungary, where students’ standardized reading and math scores fell below the national average, and fewer parents had graduated from college. Furthermore, study participation depended on teachers’ and schools’ willingness to participate; and it is possible that the included schools share certain features (e.g., a certain degree of openness) that made students more susceptible to the effects of induced proximity. Despite these potential concerns regarding external validity, which naturally arise in field experiments, we consider our findings in this particular setting promising.”

**R.2.3 Second, as regards selection on the outcome variable, my concern is that selection may be influenced by the treatment status. Just as an example, it may happen that parents whose child was not happy with the intervention because he could not establish a good bond with the deskmate, are more likely to avoid giving consent. This in turn may be more likely to happen among dissimilar pairs. For this reason, it is important to see the characteristics of these observations and make sure that parents’ decision to deny consent (or generally the missings in the outcome) is not related to treatment status.**

Thank you very much for this clarifying explanation. Such selective dropout seems possible and would indeed threaten the identification of the effect of interest (although it is not quite straightforward to gauge the impact, as the students who did not answer the questionnaire are also part of non-deskmate dyads).

We thus ran additional analyses to (1) assess the selectivity of attrition, and (2) to estimate the deskmate effect taking attrition into account. In short, we did find some evidence for selective attrition. Additional analyses that account for selective attrition in various ways (including a worst-case analysis under extreme assumptions) return somewhat smaller estimates of the deskmate effects, although the corresponding 95% credible intervals still comfortably exclude zero.

We added the following to the manuscript:

**Methods section**, p. 15, line 349: “Attrition**.** About 10% of students were omitted from our main analysis, because they did not provide friendship nominations (e.g., because they lacked parental consent for the endline survey, did not attend school on the day of the assessment, or skipped the question). Multivariate non-response models indicated some selective non-response. While gender and ethnicity did not predict missingness (p > .12), a 1 SD increase in GPA, the model predicted a 2.4 percentage point increase in the probability of response (p = .001), and a 1 SD increase in similarity (Gower’s index) predicted a small but statistically significant decrease of 1.5 percentage points in the probability of response (p = .004). To address possible bias from selective attrition, we ran two additional sets of analyses.

First, we estimated a lower bound for the deskmate effect by imputing missing friendships nominations under extremely conservative assumptions: whenever nominations were missing, we assumed that (1) the student did not nominate their deskmate and (2) the student nominated all non-deskmates who had nominated them. This minimized the number of friendships between deskmates and maximized the number of friendships between non-deskmates.

Second, we re-ran the central analyses with dyadic non-response weights. The resulting estimates identify the causal effect of interest under the assumptions that our non-response model is correctly specified. A more detailed description, the full analysis code and results of these additional analyses can be found on the Open Science Framework.”

On the OSF, we additionally provide the following details:

**Additional Analyses Attrition:** […] Second, we calculated (dyadic) non-response weights. Using a probit model that parallels our central analyses, we predicted whether or not dyads’ friendship status was missing from whether or not they were deskmates, baseline covariates (all three combinations of ethnicity, all three combinations of gender, mean dyad GPA and GPA difference), as well as the interaction between the deskmate indicator and the covariates, and classroom fixed effects. From this model, we predicted response weights, and re-ran the central analyses weighting observations with the inverse of the response probabilities. The resulting estimates identify the causal effect of interest under the assumptions that our non-response model is correctly specified. The full analysis code and results of these additional analyses can be found on the Open Science Framework.

Furthermore, the new results are reported throughout the manuscript in the respective sections:

**Results section, Deskmate Hypothesis**, p. 18, line 407: “Imputing missing outcomes in the most conservative manner results in a lower bound estimate of b = 0.17, CI_95: [0.09, 0.24]). In this model, sitting next to each other increased the probability of a manifest friendship by 4.0 percentage points (CI_95: [2.0; 6.1]), from 14.6 percent to 18.7 percent. Lastly, applying non-response weights, we estimated that the deskmate effect was b = 0.24, CI_95: [0.15, 0.33]). In this model, sitting next to each other increased the probability of a manifest friendship by 5.9 percentage points (CI_95: [3.5; 8.4]), from 14.8 percent to 20.8 percent.”

**Results section, Modification-by-Similarity Hypothesis**: Moderating Role of Overall Similarity, p. 20, line 457: Imputing missing values in the most conservative manner did not change conclusions regarding the lack of an interaction on latent friendship propensities. Furthermore, we still observed an interaction on the probability of manifest friendships (i.e., 95% credible intervals for the differences between the deskmate effects for dyads with low, average, and high similarity exclude zero), but all average marginal effects were somewhat lower and the 95% credible interval now contained zero for low-similarity dyads: $AME_{Low}=1.7$percentage points ($CI_{95}:[-0.4, 1.9]$); $AME_{Average}=3.1$ percentage points ($CI_{95}:[1.2, 5.1]$); and $AME_{High}=7.6$ percentage points ($CI_{95}:[4.0, 11.1]$). The same pattern held for analyses applying non-response weights, with average marginal effects falling between the estimates from the complete cases analysis and from the lower bound analysis: $AME_{Low}=1.1$percentage points ($CI_{95}:[-0.1, 2.7]$); $AME_{Average}=4.8$ percentage points ($CI_{95}:[2.5, 7.2]$); and $AME_{High}=10.6$ percentage points ($CI_{95}:[6.5, 15.0]$).

**Results section, modification by gender:** Imputing missing values in the most conservative manner, as well as non-response weighting, led to the same pattern of results (albeit with smaller effect estimates).

**Results section, modification by educational achievement:** Once again, imputing missing values in the most conservative manner, as well as non-response weighting, led to the same pattern of results, with overall smaller effect estimates.

**Results section, modification by ethnicity:** Imputing missing values, as well as non-response weighting, led to the same somewhat unclear pattern of results.

We believe that the results from these additional analyses warrant some qualifications to the way we present our results. We changed parts of the discussion where effect modification is discussed to accommodate the more conservative estimates.

p. 24, line 544: “Induced spatial proximity nevertheless succeeded in inducing some diverse friendships. Randomly seating boys and girls next to each other doubled their probability of nominating each other as best friends (from less than 2 to 4 percentage points). The intervention also substantially increased friendships between students with strong and weak baseline GPAs (from 11 to 17 percentage points). Finally, whether or not seating Roma and non-Roma students next to each other increased friendships across ethnic lines remained unclear in our data; the estimate was beset with statistical uncertainty due to relatively small numbers of Roma students in the sample and sensitive to assumptions about missing data.”

**R.2.4. related to the above point and to the identification of the effect: I was asking about pre-existing friendships because it is more likely that, within a class, pupils tend to befriend similar peers. Thus, the strongest effect for similar peers may be due to higher likelihood of a pre-existing bond. Since the authors do not have such information, they should acknowledge this caveat when describing their design and above all their results.**

Thank you for this helpful clarification. We initially thought that the reviewer was concerned about the *causal identification* of the deskmate effect; we now believe that the reviewer is wondering about the mechanism that may explain *effect modification* (effect heterogeneity) of the deskmate effect by similarity. We apologize for our earlier misunderstanding.

The reviewer’s hypothesis strikes us as plausible.

Suppose, for example, that the intervention of being seated next to each other may be highly effective in preventing the dissolution of existing friendships, but less effective in inducing new friendships. If similar students are more likely to have pre-existing friendships, then our finding that sitting next to each other, on net, increases the probability of friendship more among similar dyads than among dissimilar dyads could be explained by the greater probability of pre-existing friendships among similar dyads. Assuming that this reasoning describes the actual data-generating mechanism, then pre-existing friendships would be a mediator of the causal effects of similarity on friendship at endline.

This account does not impinge on the causal interpretation of the deskmate effect (either of the average effect, or of the subgroup effects conditional on baseline similarity). We struggled to understand this particular point because we started from the premise that our design does not allow us to causally identify the effects of similarity (as explained in the Method section). But of course, the term “effect modification” may still be evocative of certain types of causal stories. To avoid any such misinterpretation, we re-read the discussion section, took greater care when communicating the effect modification issue, and also included the possible and plausible interpretation suggested by the reviewer.

p. 23, line 531: “But since the effect of a given increase in the latent propensity toward friendship on the formation of a manifest friendship also depends on the dyad’s baseline propensity toward friendship, and since more similar dyads have a greater baseline propensity toward friendship (homophily), the intervention was more successful among similar students than among dissimilar students. One potential explanation could be that being seated next to each other may be particularly effective at preventing the dissolution of pre-existing ties (as compared to inducing new ties), which are more prevalent among similar dyads; our design, however, does not allow for the identification of different possible mechanistic explanations. The three dimensions of similarity that we investigated contributed to the overall pattern to varying degrees:…”

**R.2.5. I better explain the question in R.2.6 (I apologise for the mistake, it was line 169): the authors state “94.4 percent of the dyads in which students actually sat next to each after the second week of classes comprised students who were supposed to sit next to each in the intended seating chart” (line 180). Given that the authors have information on compliance, are the results robust (stronger?) if the authors exclude the 5.6% (100-94.4) of the dyads who were not compliant. What if they exclude also the dyads in which students did not actually sat next to each?**

Thank you for this clarification! We re-ran analyses excluding the deskmate dyads who did not adhere to the treatment (i.e., dyads who were assigned to sit next to each other but didn’t do so, and dyads who were not assigned to sit next to each other but did do so).

The resulting estimates were indeed stronger than the results of the main pre-reported analysis reported in the manuscript. In the analysis that excluded these dyads, students had a 22.6% probability of being friends with a deskmate (vs. 22.3% in our main analysis) and a 15.3% probability of being friends with a non-deskmate (vs. 15.3%), the average marginal effect of being seated next to each other was 7.2 [4.9; 9.8] percentage points (vs. 7.0, [4.6; 9.4]).

We now briefly mention these additional results in the main body of the text:

p. 18, line 399: “Excluding dyads who did not adhere to treatment resulted in a slightly larger effect estimate of 7.2 percentage points ($CI_{95}$: [4.9; 9.8]).”

**R.2.6. I suggest again to spellcheck the paper: line 207 “1if”; line 512 “the transformations of latent propensities into manifest friendships remains”**

Thank you very much for carefully reading the manuscript and catching these typos. We again spellchecked the whole manuscript and caught two superfluous whitespaces.
